# Deciphering the Antibacterial Role of Peptide From *Bacillus subtilis* subsp. *spizizenii* Ba49 Against *Staphylococcus aureus*

**DOI:** 10.3389/fmicb.2021.708712

**Published:** 2021-08-19

**Authors:** Ramita Taggar, Sanpreet Singh, Vijayender Bhalla, Mani Shankar Bhattacharyya, Debendra K. Sahoo

**Affiliations:** ^1^CSIR-Institute of Microbial Technology, Sector 39A, Chandigarh, India; ^2^Academy of Scientific and Innovative Research, New Delhi, India

**Keywords:** antimicrobial peptides, *Staphylococcus aureus*, biofilm, intracellular activity, ROS, PAE, scratch assay

## Abstract

An increase in antibiotic resistance has led to escalating the need for the development of alternate therapy. Antimicrobial peptides (AMPs) are at the forefront of replacing conventional antibiotics, showing slower development of drug resistance, antibiofilm activity, and the ability to modulate the host immune response. The ESKAPE (*Enterococcus faecium*, *Staphylococcus aureus*, *Klebsiella pneumoniae*, *Acinetobacter baumannii*, *Pseudomonas aeruginosa*, and *Enterobacter* species) pathogens that jeopardize most conventional antibiotics are known to be involved in severe respiratory tract, bloodstream, urinary tract, soft tissue, and skin infections. Among them, *S. aureus* is an insidious microbe and developed resistance against conventional antibiotics. In the present study, an AMP (named as peptide-Ba49) isolated from *Bacillus subtilis* subsp. *spizizenii* strain from *Allium cepa* (the common onion) exhibited strong antibacterial efficacy against *S. aureus* ATCC 25923. The mode of action of this peptide-Ba49 on *S. aureus* was deciphered through various sensitive probes, i.e., DiSC_3_ (5) and H_2_DCFDA, suggesting the peptide-Ba49 to be acting upon through change in membrane potential and by triggering the production of reactive oxygen species (ROS). This induced disruption of the cell membrane was further supported by morphological studies using scanning electron microscopy (SEM). Investigations on a possible post-antibiotic effect (PAE) of peptide-Ba49 showed prolonged PAE against *S. aureus*. Furthermore, the peptide-Ba49 prevented the formation of *S. aureus* biofilm at low concentration and showed its potential to degrade the mature biofilm of *S. aureus*. The peptide-Ba49 also exhibited intracellular killing potential against *S. aureus* ATCC 25923 in the macrophage cells, and moreover, peptide-Ba49 was found to bolster the fibroblast cell migration in the scratch assay at low concentration, exhibiting a wound healing efficacy of this peptide. These studies demonstrated that peptide-Ba49 isolated from the strain *B. subtilis* subsp. *spizizenii* could be a therapeutic candidate to combat the pathogenic *S. aureus* infections.

## Introduction

The expeditious emergence of bacterial resistance toward conventional antibiotics, especially those related to staphylococcal infections, has become a serious healthcare concern worldwide (Mohamed et al., 2016). *Staphylococcus aureus* is an opportunistic pathogen found in humans and animals and is a major cause of morbidity and mortality in the community- and hospital-acquired infections (Gresham et al., 2000). Generally, *S. aureus* is considered an extracellular pathogen, but its invasion ability plays a critical role in cases of pertinacious and chronic infection (Clement et al., 2005). Also, it has the capability of causing skin and soft tissue infection, sepsis, mastitis, urinary tract infection, endocarditis, bone and joint infections, food poisoning, biofilm-associated infections, or septicemia (Lowy, 1998; Tong et al., 2015; Rollin et al., 2017). Infections like bacteremia and skin abscesses are generally caused by planktonic *S. aureus* cells by producing secreted toxins and exo-enzymes, whereas chronic infections are associated with *S. aureus* biofilm (Gordon and Lowy, 2008). This organism attaches and recurs on host tissues such as bone and heart valves causing osteomyelitis and endocarditis, respectively, and also on implanted materials like pacemakers, prosthetic joints, etc. (Parsek and Singh, 2003; Kiedrowski and Horswill, 2011; Barrett and Atkins, 2014; Chatterjee et al., 2014). During the implantation of medical devices or biomaterials within the host, the host proteins such as fibronectin, fibrinogen, or fibrin coat these implants and become a potential target of interaction with the matrix-binding proteins present on the surface of *S. aureus*, ultimately leading to the formation of biofilm on these implants (Cheung and Fischetti, 1990; Francois et al., 1996).

In nature, bacteria generally dwell a biofilm, a complex and dynamic surface-associated community. These are sessile microbial consortia that establish a three-dimensional structure (Costerton et al., 1999). These communities of microbes adhere to various surfaces and are confined in a self-produced extracellular matrix (Monroe, 2007). The biofilm growth displays the altered physiologies with respect to the expression of a gene and the production of proteins (Parsek and Singh, 2003; Archer et al., 2011; Kiedrowski and Horswill, 2011). *Staphylococci* are associated with the most persistent cause of biofilm-associated infections and present as a commensal bacterium on human and other mammalian mucous and skin surfaces (Vuong and Otto, 2002). The staphylococcal biofilms are developed in three main stages, i.e., (i) initial adhesion to the surface, (ii) biofilm formation and maturation, and (iii) dispersal and their maturation are linked to the EPS production, which allows the biofilm stabilization (Lister and Horswill, 2014). The dispersion phase related to the acquittal of cells that colonize the new sites.

Intriguingly, it has been observed that mature biofilm dynamically changes the niche at the site of formation, which ultimately prompts the survival of dormant and non-dividing cells within this biofilm and hampers the antibacterial efficacy of conventional antibiotics against it (Galdiero et al., 2019). Moreover, the cells of biofilm are less susceptible and show profound resistance against various available drugs and antimicrobial agents as compared to planktonic cells. There are various mechanisms that make the biofilm cell highly resistant to antibacterial agents, such as (i) reduction in growth and metabolic activity of cells residing deep in biofilm; (ii) the biofilm matrix, i.e., EPS, which acts as an adsorbent and limits the availability of antibiotics to the cells present within the biofilm; and (iii) biofilm cells protecting themselves using mechanisms such as multidrug efflux pumps and regulons (Brown et al., 1988; Gilbert et al., 2002). Generally, *S. aureus* is considered as an extracellular organism. However, it can survive and persist within the phagocytotic and non-phagocytotic cells and is responsible for frequent cause and relapsing of infections (Lemaire et al., 2007). The intracellular *S. aureus* escapes the extracellular host antibacterial defense mechanism and antibiotics have the tendency to infect other cells, and thus, it becomes more challenging to treat the infections (Mohamed et al., 2014).

Most drugs are poorly diffused into phagocytes or do not take up by the same intracellular compartment as the bacteria; thus, their antibacterial activity may be affected by the intracellular environment or by the change in bacterial metabolism (Brinch et al., 2009). Antibiotics like oxacillin, moxifloxacin, and levofloxacin etc., show poor intracellular penetration and are affected by low cellular level of accumulation (Wang et al., 2018). Antibiotics such as linezolid and gentamicin are partially and inconsistently active due to the acidic environment because of aminoglycosides and intra-lysosomal constituent binding (Van Der Auwera et al., 1991). This may lead to the use of some antibiotics at a higher extracellular concentration to get a significant activity, which further causes drug resistance and side effects. Thus, these problems have aggrandized the need for an improved or alternative therapeutics (Wang et al., 2018).

In recent years, antimicrobial peptides (AMPs) are at the forefront as alternatives to conventional antibiotics to reduce the effectiveness of this pathogenic infection. These AMPs are having multiple modes of actions such as membrane permeabilization, protein synthesis inhibition, and DNA binding etc., (Wimley, 2010; Wang et al., 2019). AMPs, produced by all living organisms, are small molecules (10–100 amino acids) that play an essential role in innate immunity (Di Somma et al., 2020). Naturally produced AMPs play a key role in host defense systems in prokaryotes and eukaryotes and act as weapons to fight against various pathogens (Yeaman and Yount, 2003; Ageitos et al., 2017). In addition to antimicrobial activity, these AMPs also represent a promising therapeutic option against biofilm infections (Lombardi et al., 2015). Furthermore, AMPs also deploy intracellular inhibitory activities as a supportive mechanism to achieve efficient killing (Le et al., 2017). There are various sources from which an AMP can be isolated, such as humans, animals, plants, bacteria, and fungi etc., (Yang et al., 2016; Huang et al., 2017; Huan et al., 2020). Among microorganisms, the genus *Bacillus* is a vast arsenal of antimicrobials and a promising host for screening new bioactive compounds (Lajis, 2020; Taggar et al., 2021).

Previously, we had reported screening and isolation of strain Ba49, i.e., *Bacillus subtilis* subsp. *spizizenii*. The peptide-Ba49 from this strain was successfully purified and characterized. The peptide-Ba49 showed a molecular weight of 3,319.2 Da and was characterized as subtilin on the basis of *de novo* sequencing and antiSMASH results of whole genome of strain Ba49 (Taggar et al., 2021). Furthermore, peptide-Ba49 was found to be stable in a wide range of temperature and pH and showed low minimum inhibitory concentration (MIC) value against various *S. aureus* and notorious methicillin-resistant *S. aureus* (MRSA) strains, i.e., between 0.5 to 16 μM. Following 4 h of peptide-Ba49 treatment of *S. aureus* cells at 2 × MIC, a 5–6 log reduction of *S. aureus* cells was observed and this was further confirmed by using propidium iodide (PI) staining and transmission electron microscopy (TEM) studies. The above study also showed a low cytotoxicity effect against the mammalian cell lines at a concentration much higher than that of its MIC values (Taggar et al., 2021). Based on the above results, the present study was undertaken to explore the role of the peptide-Ba49 in inhibiting the formation of *S. aureus* ATCC 25923 biofilm and also in eradication of pre-formed biofilm. Furthermore, the post-antibiotic effect (PAE) of peptide-Ba49 against *S. aureus* planktonic cells at different time intervals and its intracellular killing efficacy against *S. aureus* were investigated. The fibroblast cell migration capability of peptide-Ba49, which putatively contributes to wound healing, was also evaluated.

## Materials and Methods

### Bacterial Strains and Cell Line

The bacterial strain *B. subtilis* subsp. *spizizenii* MTCC 13006 was used for the production of peptide-Ba49 (Taggar et al., 2021). *S. aureus* (ATCC 25923) was used as a model organism to decipher the role of peptide-Ba49. RAW 264.7 cell line (ATCC^®^ TIB-7) and L929 cell line (ATCC^®^ CCL-1) were used to study the intracellular killing activity of peptide-Ba49 in macrophage and for cell migration assay, respectively.

### Reagents

Muller-Hinton Broth (MHB), Muller-Hinton Agar (MHA), Nutrient Broth (NB), and crystal violet (CV) were purchased from HiMedia, India. Roswell Park Memorial Institute-1640 Medium (RPMI-1640) was procured from Sigma-Aldrich, United States. PI and SYTO 9 were purchased from Invitrogen (Thermo Fisher Scientific, India). 2,7-dichlorodihydrofluorescein diacetate (H_2_DCFDA), 3,3-dipropylthiacarbocyanine [DiSC_3_ (5)], XTT sodium3′-[1-[(phenylamino)-carbony]-3,4-tetrazolium]-bis(4-methoxy-6-nitro) benzene-sulfonic acid hydrate, and calcofluor white M2R (CFW) were procured from Sigma-Aldrich, United States. Peptide-Ba49 used in all studies was produced by *B. subtilis* subsp. *spizizenii* MTCC 13006 using ZMB medium. Following harvest at 36 h, the peptide was extracted from fermentation broth using Diaion HP-20 resin followed by further purification using a series of chromatographic techniques, i.e., ion-exchange chromatography and RP-HPLC. Furthermore, the purity of purified peptide was confirmed through Tricine SDS PAGE and RP-HPLC (as single band and single active peak) as reported earlier (Taggar et al., 2021).

### *In vitro* Antibacterial Activity of Peptide-Ba49 Against *S. aureus* ATCC 25923

#### Post-antibiotic Studies

The post-antibiotic studies were performed according to Tambat et al. (2019) with some modifications. *S. aureus* cells (∼10^5^ CFU/ml) were treated with the two different concentrations, 4 μM (1 × MIC) and 8 μM (2 × MIC), of peptide-Ba49 in MHB medium. Followed by incubating the samples in a shaking incubator at 37°C and 150 rpm for 2 and 4 h, the samples were centrifuged and the pellets were further diluted after adjusting to the same cell density with fresh MHB medium in the ratio of 1:100 to minimize the effect of the peptide and transferred to 96-well plates. For studying the growth kinetics, the plates were incubated at 37°C and low shaking (infinite M plex TECAN) and the cell O.D._600_ was measured at an interval of 30 min up to 12 h. The cells without any treatment were taken as positive control and processed similarly. The experiments were carried out in duplicate and cell growth at O.D._600_ was measured in triplicate for each experiment.

#### Intracellular Killing Efficacy of Peptide-Ba49 in RAW 264.7 Cells

The intracellular activity of peptide-Ba49 was determined by seeding the RAW 264.7 macrophages (5 × 10^5^cells/ml) into six-well plates containing RPMI-1640 and 10% FBS (without antibiotic) and incubating the plates in a carbon dioxide (CO_2_) incubator at 37°C for 24 h. Later, the macrophages were infected with *S. aureus* using a 1:10 multiplicity of infection (MOI) in RPMI-1640 supplemented with 10% FBS (without antibiotic) for 2 h. After infection, 50 μg/ml of gentamicin was added to each well and the plates were incubated for 30 min to eliminate the extracellular bacteria. Furthermore, the macrophages were washed twice with 1 × PBS, followed by the treatment with different concentrations of peptide-Ba49, i.e., 4 μM (1 × MIC) and 8 μM (2 × MIC). Additionally, nisin in two different concentrations, i.e., 4 μM (1 × MIC) and 8 μM (2 × MIC), was taken as positive control and cells without any treatment were taken as negative control. Later, the cells were washed with 1 × PBS and then lysed with 0.1% saponin. The numbers of intracellular bacteria were measured at 12 and 24 h by colony counting. The experiment was carried out in triplicate with two individual repeats.

#### Studies on Mechanism of Action of Peptide-Ba49 Against *S. aureus* ATCC 25923

The mechanism of peptide-Ba49 against *S. aureus* was studied using fluorescence spectroscopy and scanning electron microscopy (SEM) as described in the following paragraphs.

#### Reactive Oxygen Species (ROS) Assay

The ROS production by *S. aureus* after the treatment with peptide-Ba49 was determined by using sensitive probe 2,7-dichlorodihydrofluorescein diacetate (H_2_DCFDA) fluorescent dye (Sigma-Aldrich), which could detect a broad range of ROS including nitric oxides and hydrogen peroxides (Arakha et al., 2015). *S. aureus* cells at concentration of 5 × 10^4^ CFU/ml were treated with peptide-Ba49 at 4 μM (1 × MIC) and 8 μM (2 × MIC) for the sub-lethal stage (2 h). This was followed by pelleting the cells by centrifugation, washing the pellets with 1 × PBS, and transferring the cells to 96-well plates. Furthermore, the plates containing cells were mixed with H_2_DCFDA at a final concentration of 5 μM and incubated at 37°C for 1 h. Untreated cells were taken as control whereas cells mixed with polymyxin B (60 μg/ml) was taken as a positive control and the fluorescence was measured at an excitation and emission of 485 and 525 nm, respectively. The experiment was done in triplicate, each with three individual repeats.

#### Cytoplasmic Membrane Disruption Assay

A modified method (Kwon et al., 2019) based on the membrane potential-sensitive dye, DiSC_3_ (5), was used to evaluate the effect of peptide-Ba49 on the membrane disruption of *S. aureus* cells. Briefly, following the overnight culture of *S. aureus* in MHB medium at 37°C, the cells were washed with 5 mM HEPES buffer containing 20 mM glucose and resuspended to an O.D_600_ of 0.05 in 5 mM HEPES buffer with 20 mM glucose and 100 mM KCl. Furthermore, DiSC_3_ (5) was added at a final concentration of 0.4 μM to each well of 96-well plates and incubated for 1 h. The fluorescence was allowed to be stable and then peptide-Ba49 at concentrations of 4 μM (1 × MIC) and 8 μM (2 × MIC) was added to each well separately. The cells without any treatment were taken as control whereas the cells treated with polymyxin B were taken as a positive control. Following 1 h of incubation, fluorescence of the cells was measured at an excitation and emission of 622 and 670 nm, respectively. The experiments were carried out in triplicate with two independent repeats.

### Scanning Electron Microscopy

SEM was used to examine the structural changes of *S. aureus* treated with peptide-Ba49. *S. aureus* cells at a concentration of 1 × 10^7^ CFU/ml in the NB medium was mixed with 4 μM (1 × MIC) of purified peptide Ba-49 and incubated at 37°C for 240 min. Cells not treated with the purified AMP was taken as a control. Both peptide-Ba49 treated and untreated *S. aureus* cells were centrifuged at 5,000 rpm and 4°C for 10 min and washed twice with 1 × PBS (0.1 M, pH 7.4). Poly(L-lysine)-coated coverslip was used for cell attachment. Cells were then fixed on these coated coverslips with Karnovsky’s fixative for 2 h at 4°C followed by twice washing with 1 × PBS. These cells were then dehydrated with gradients of ethanol (30, 50, 70, 90, and 100%) each for 30 min (David et al., 1973). Then, the cells were air-dried, coated with platinum, and observed under the SM-IT 300 LV scanning electron microscope (JEOL, Tokyo Japan).

#### *S. aureus* Biofilm Formation and Its Assessment

A static microtiter plate assay was performed to check the ability of the test strain to form biofilm. Briefly, the strains of *S. aureus* were grown overnight in LB supplemented with 1% (w/v) sucrose (LBS) at 37°C. Later, the cells were diluted to 1:100 in fresh LBS media and 100 μl of this diluted culture was inoculated into each well of a sterile 96-well microtiter plate. Additionally, 100 μl of LBS was used as a negative control followed by incubation of the plate at 37°C for 24 h to allow for biofilm formation (Shikha et al., 2020).

A variety of direct and indirect methods for quantification of cells in biofilms have been reported (Wilson et al., 2017). A modified method of O’toole (2011), based on the CV staining of the biofilm mass, was used (O’toole, 2011) for the determination of biofilm biomass. Following the removal of biofilm plates, the biofilm was washed gently with the 1 × PBS to remove the planktonic cells. Later, the remaining biofilm cells were fixed by adding 200 μl of 100% methanol into each well. Following incubation at room temperature for 15 min, the remaining methanol was aspired and the plate was air-dried for 15–20 min. Once fixed, biofilm was stained with 200 μl/well of 0.1% CV and the plates were incubated for 15 min at room temperature. Later, the stained plates were washed twice with distilled water to remove the extra stain followed by drying of the plates. This was followed by preparation of the biofilm suspension by adding 30% acetic acid and leaving the suspension at room temperature for 30 min to dissolve the stain properly. The absorbance was measured at 595 nm and the values obtained were considered as adhere biofilm index to the surface of the well and the extracellular mass produced by them. The percentage of biofilm was calculated by using a previously described formula (Shikha et al., 2020):

%biofilm=(ODo595ftreatedcells/ODo595funtreatedcells)*100.

In the present study, XTT sodium3′-[1-[(phenylamino) -carbony]-3,4-tetrazolium]-bis(4-methoxy-6-nitro) benzene-sulfonic acid hydrate (Sigma-Aldrich, United States) was used to estimate the viability of biofilm (Haney et al., 2018). In this method, following the formation of the *S. aureus* biofilm (as describe above), the media was gently removed and the biofilm was washed with 1 × PBS to remove the planktonic cells. Later, the aspired cells were dissolved in 100 μl of 1 × PBS, followed by the addition of 30 μl of 1% sucrose-containing XTT-menadione solution and incubation in the dark at 37°C for 1–2 h. The absorbance was measured at 490 nm, which reflected the viability of the cells present in the biofilm (Shikha et al., 2020) and % viability of biofilm was estimated as follows:

%viabilityofbiofilm=(ODo490ftreatedcells/ODo490funtreatedcells)*100.

#### Evaluation of Inhibitory Effect of Peptide-Ba49 on *S. aureus* Biofilm Formation

The inhibitory effect of peptide-Ba49 on *S. aureus* biofilm formation was measured by taking 1:100 diluted *S. aureus* cells (in LBS medium) in a microtiter plate and adding different concentrations of peptide-Ba49 (2, 4, 8, and 16 μM) to each well separately at the beginning of the assay. The plates were incubated at 37°C for 24 h. The untreated cells were taken as control. Following incubation, the plate was removed and gently washed with 1 × PBS and stained with 0.1% CV as described above. Optical density was measured at 595 nm to determine the final biomass of the biofilm. Data obtained in triplicate were analyzed and expressed as the mean plus standard deviation. The experiment was performed in three biological repeats.

The viability of the cells within the biofilm was measured using the XTT method as described above with the following modifications. Following the formation of biofilm in LBS media containing different concentrations of peptide-Ba49 (2, 4, 8, and 16 μM), the plates were removed and washed with 1 × PBS. XTT was added as described above and incubated in the dark for 1–2 h. The absorbance of the cell suspension was measured at 490 nm to determine the presence of viable cells in the biofilm. Data obtained in triplicate were analyzed, and the cell viability was expressed as the mean plus standard deviation. The experiment was performed in three biological repeats.

#### Confocal Laser Scanning Microscopy (CLSM) Imagining of *S. aureus* Biofilm

The effect of peptide-Ba49 on *S. aureus* biofilm formation was analyzed using confocal laser microscopy (CLSM). A previously described method (Haque et al., 2016) with some modifications was used for *S. aureus* biofilm formation. The *S. aureus* cells were grown overnight in LBS medium at 37°C and later the cells were diluted to 1:100 in fresh LBS medium. One thousand microliters of this diluted culture was poured into each 12 well-plate containing sterile coverslips and was further treated with 16 μM of peptide-Ba49, followed by incubation at 37°C for 24 h in a static condition. Cells without peptide treatment were taken as a control. The media was gently decanted after incubation, and the wells were washed twice with 1 × PBS to remove the planktonic cells. Three fluorescent dyes, i.e., SYTO 9, PI, and Calcofluor white M2R (CFW), were used for staining of the biofilm. Working solutions of SYTO 9, PI, and Calcofluor white M2R (CFW), in concentrations of 5, 30, and 0.15 μM, respectively, were prepared in 1 × PBS. The biofilms were stained with 500 μl of working solution of SYTO 9 (5 μM) and PI (30 μM) for 15 min in the dark, followed by staining with 500 μl of working solution of CFW (0.15 μM) for 3 min in the dark. Following staining, the coverslips were washed with sterile water to remove the extra stain and then placed over the glass slide with a drop of mounting oil in between to avoid the dryness and sealing the coverslips from any air contact. The stained biofilm was observed under CLSM with 40 × numerical aperture (NA) and excitation at 488 and 543 nm. Emissions at 495–535 nm (green color) and 580–700 nm (red color) were characterized for viable bacteria and dead bacteria, respectively. The EPS of biofilm when excited at 405 nm emitted light at 413–480 nm (blue color) (Hou et al., 2018).

#### Eradication of *S. aureus* Biofilm

Formation of *S. aureus* biofilm was carried out as previously described. Once the biofilm was established (24 h), it was gently washed with 1 × PBS to remove the planktonic cells and then mature biofilm was treated with different concentrations of peptide, i.e., 8, 16, 32, and 64 μM, and incubated at 37°C for 24 h. Following incubation, the plates were gently washed with 1 × PBS and then stained with 0.1% CV as described before. The absorbance was taken at 595 nm to determine the final biofilm biomass. Data obtained in triplicate were analyzed and expressed as the mean plus standard deviation. The experiment was performed in three biological repeats.

The viability of the cells within the pre-formed biofilm after peptide-Ba49 treatment was determined using the XTT method as described before. Briefly, following the treatment of biofilm with peptide-Ba49 at concentrations of 8, 16, 32, and 64 μM, the plates were gently washed with 1 × PBS, XTT was added, and the plates were incubated in the dark for 1–2 h. The absorbance was measured at 490 nm to determine the presence of viable cells within the pre-formed biofilm. Data obtained in triplicate were analyzed and expressed as the mean plus standard deviation. The experiment was performed in three biological repeats.

### Cell Migration Assay of Peptide-Ba49

The cell migration capacity of purified peptide-Ba49 was carried out by performing *in vitro* cell migration studies of L929 cells (Balekar et al., 2012). A cell density of 5 × 10^5^ cells/well were seeded into a six-well plate containing RPMI-1640 culture medium supplemented with 10% FBS and incubated overnight in a humidified carbon dioxide incubator at 37°C and 5% CO_2_ to allow the cells to adhere on the surface. After incubation, the media was decanted and the adherent layer was scratched, followed by the removal of cellular debris by washing with 1 × PBS (pH 7.2). Later, fresh RPMI media supplemented with 10% FBS was added followed by the addition purified peptide-Ba49. Untreated cells were taken as a control. The cells were then incubated in a humidified carbon dioxide incubator at 37°C and 5% CO_2_. The scratch area images were captured using a 40 × magnification microscope at different intervals of 0, 12, 24, and 36 h.

#### Statistical Analysis

The data were statistically analyzed using one-way analysis of variance (ANOVA) with Dunnett’s multiple comparison tests unless otherwise mentioned using GraphPad Prism 7 software. ^∗^*p* < 0.05, ^∗∗^*p* < 0.01, ^∗∗∗^*p* < 0.001, ^****^p < 0.0001, and ^*****^p < 0.00001 were considered significant and *p* > 0.05 was considered as non-significant (ns).

## Results

### Pharmacodynamic Studies of Peptide-Ba49 Against *S. aureus*

#### PAE of Peptide-Ba49

**FIGURE 1:**
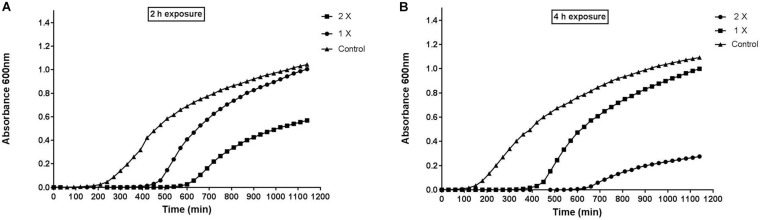
Post-antibiotic effect of peptide-Ba49. **(A)** Post 2 h and **(B)** post 4 h of exposure of peptide-Ba49. The bacteria showed suppression for approximately 7 and 10 h at post 2 and 4 h exposure to peptide-Ba49 at a concentration of 1 × MIC and 2 × MIC, respectively. The bacterial repair was also reduced at post 4 h exposure to peptide-Ba49 at a concentration of 2 × MIC.

PAE is a phenomenon that describes the delayed regrowth of bacteria following treatment with any antimicrobial agent. It is one of the characteristics of pharmacodynamics being increasingly applied to the design of dosing regimens of antimicrobial agents. The inhibition in growth of the pathogen is followed by recovery and regain of their infectious potential, once the efficacy of antimicrobial agent was finished/reduced (Saravolatz et al., 2017). Figure 1 suggested that *S. aureus* growth was delayed post 2 and 4 h treatment at two different concentrations of peptide-Ba49. Interestingly, peptide-Ba49 at 1× and 2× MIC was found to suppress the growth of pathogen by approximately 7 and 10 h compared to control (no treatment). However, in the case of 4 h post-exposure at 2× MIC, the re-growth of the *S. aureus* cells was observed to be less as compared to post 2 h treatment at 2× MIC. These results indicated that higher cellular damage occurred within the *S. aureus* cells following 4 h of treatment of cells at 2× MIC of peptide-Ba49.

#### Intracellular Killing Efficacy of Peptide-Ba49 Against *S. aureus* in Macrophage Cell Line

**FIGURE 2:**
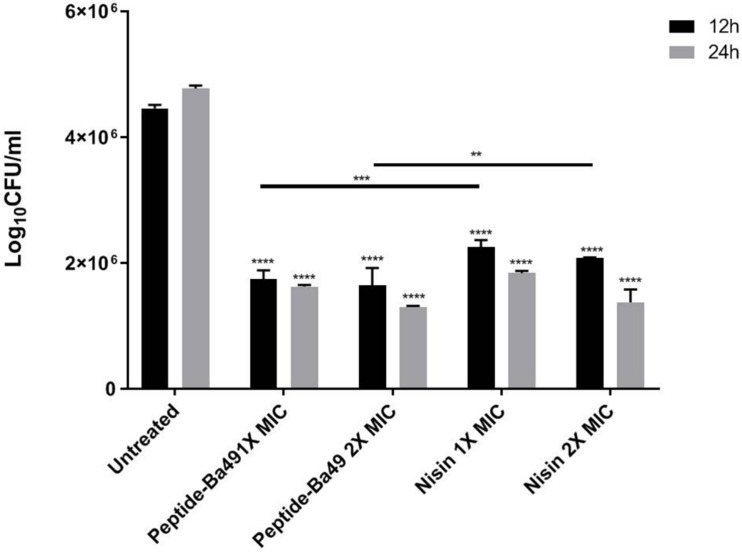
Intracellular activity of peptide-Ba49 against *S. aureus*-infected RAW 264.7 cells: The figure showed the intracellular killing efficacy of peptide-Ba49 against *S. aureus* ATCC 25923 infected macrophage cells. After 24 h of incubation with the peptide-Ba49, there was a significant reduction in the log_10_ CFU/ml, as compared to the untreated one. The analysis was carried out in two-way ANOVA with Tukey’s multiple comparisons test. ****p < 0.0001, ***p < 0.0001, **p < 0.01 indicated the significant difference. The values “mean ± SD” were representative of two independent experiments done in triplicate.

The intracellular activity of peptide-Ba49 was evaluated by infecting the macrophage cell line, i.e., RAW 264.7 with *S. aureus*. Macrophage cells are one of the first line of defense innate cells that came into action during any infection in the host. It was observed that upon treating the infected macrophages with two different peptide concentrations, i.e., 4 μM (1 × MIC) and 8 μM (2 × MIC) for 12 and 24 h, there was a decline in log_1__0_CFU/ml. Intriguingly, there was significant decrease in bacterial burden in macrophages treated with 4 μM (*p* < 0.0001) and 8 μM (*p* < 0.0001) as compared to untreated cells at 12 h; a similar reduction in bacterial count was also observed in case of 24 h treated cells with 4 μM (*p* < 0.0001) and 8 μM (*p* < 0.0001). Also, there were significant declines in bacterial count in cases of *S. aureus* cells treated with peptide-Ba49 (1 × MIC and 2 × MIC) (*p* < 0.001, *p* < 0.01) as compared to positive control (cells treated with nisin at 1 × MIC and 2 × MIC). It depicted peptide-Ba49 to be having very potent killing activity against intracellular pathogens hidden and surviving inside the host cell. Furthermore, it also demonstrated the peptide-Ba49 to be having significantly higher antibacterial potency as compared to well-known AMPs such as nisin (Figure 2).

#### Mechanism of Action of Peptide-Ba49 Against *S. aureus*

**FIGURE 3:**
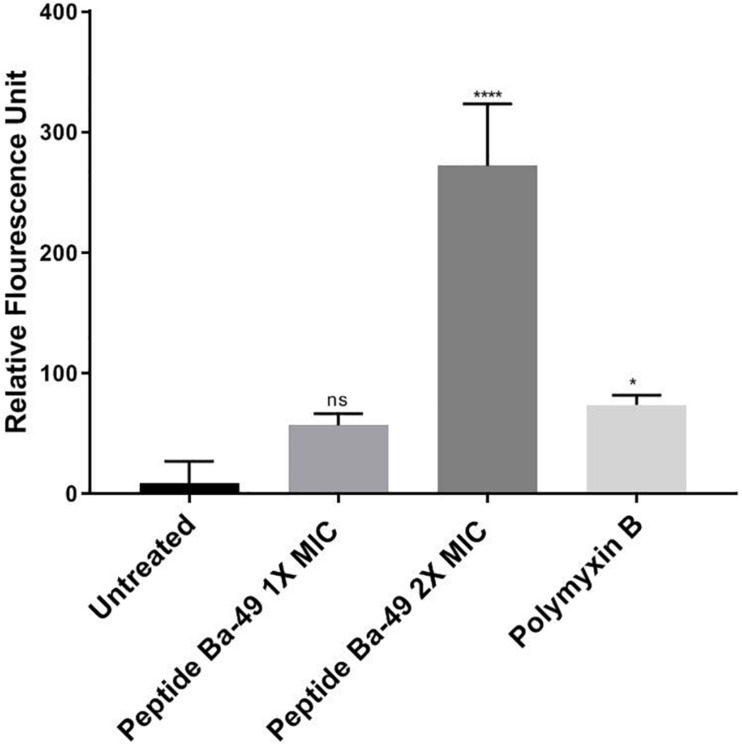
Spectrometric analysis of ROS level alteration in *S. aureus*. Following treatment of *S. aureus* cells with varied concentrations of peptide-Ba49, the cells were stained with ROS marker 2,7-dichlorodihydrofluorescein diacetate (H_2_DCFDA). The histogram represents fluorescence of H_2_DCFDA dye normalized with untreated cells. One-way ANOVA followed by Holms–Sidak multiple comparisons test showed *p < 0.05 significant difference between polymyxin B and the untreated one. Intriguingly, we observed a significant difference at 2 × MIC (with ****p < 0.0001) as compared to untreated.

**FIGURE 4:**
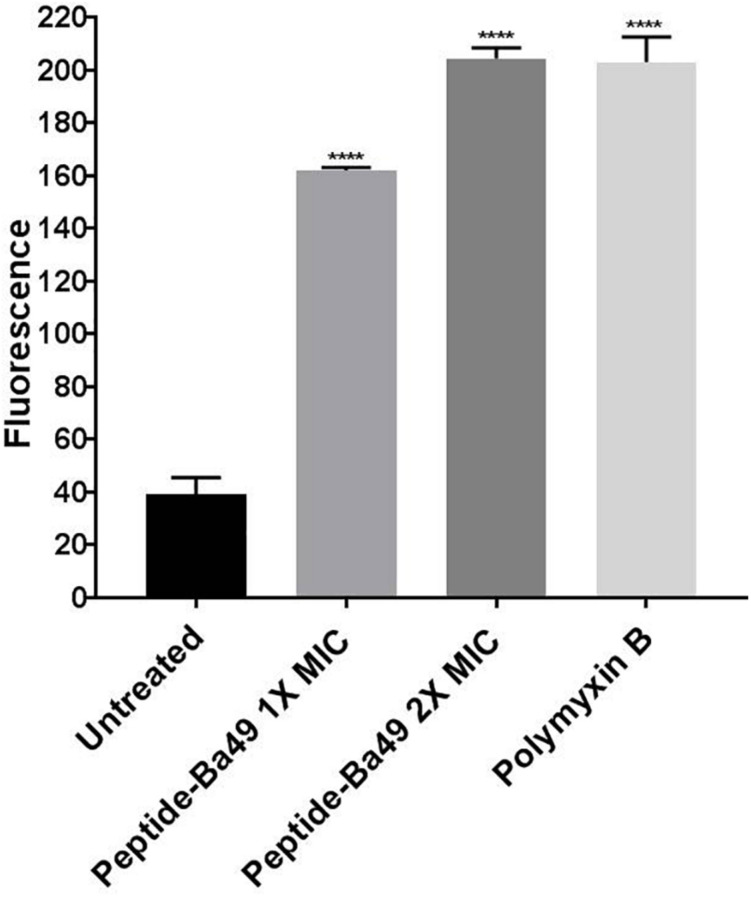
Spectrometric analysis of membrane potential of *S. aureus* cells. Cell membrane permeability of *S. aureus* ATCC 25923 was evaluated with the release of voltage-sensitive dye DiSC_3_-(5) during peptide-Ba49 treatment. Fluorescence was measured spectroscopically at 620–670 nm excitation and emission wavelengths. *S. aureus* cells were treated with peptide-Ba49 at concentrations of 4 μM (1 × MIC) and 8 μM (2 × MIC). Significant increase in fluorescence signal was observed in peptide-treated cells as compared to untreated cells. Polymyxin B-treated cells were taken as a positive control. The analysis was carried out in two-way ANOVA with Dunnett’s multiple comparisons test. ****p < 0.0001 indicated the significant difference between control and treatment groups. The values “mean ± SD” were representative of two independent experiments done in triplicate.

In case of the healthy untreated bacterial cell, ROS production is a natural side effect of aerobic respiration. The bacteria can produce enzymes like catalase and superoxide dismutase to prevent damage, which further detoxifies the ROS (Greenberg and Berger, 1989; Gasser et al., 2016). For determining the effect of the peptide on the enhancement of ROS production, *S. aureus* was treated with different concentrations of peptide in the presence of H_2_DCFDA, an unspecific probe for ROS. It showed that the ROS production was enhanced when treated with 8 μM (2 × MIC) of peptide than control, whereas, at a peptide concentration of 4 μM (1 × MIC), ROS production was low (Figure 3). This suggested that an increase in the concentration of peptide resulted in an enhancement of ROS production, which indirectly affected the growth of *S. aureus* ATCC 25923. However, it is known that if the bacterial cells fail to minimize the excess of intracellular ROS production, membrane depolarization occurs, which ultimately leads to cell death (Yadav et al., 2020; Zhou et al., 2020). The same phenomenon was observed in our study. The effect of peptide-Ba49 on the membrane potential of *S. aureus* was studied by determining the localization of a voltage-sensitive fluorescent cationic probe DiSC_3_ (5) in *S. aureus* cells. Due to its cationic nature, DiSC_3_ (5) accumulates on the polarized membranes, which results in self-quenching of fluorescence. However, during membrane depolarization, de-quenching of fluorescence dye has been reported (Boix-Lemonche et al., 2020). After treating *S. aureus* cells with peptide-Ba49 for 1 h, an increase in fluorescence was observed compared to that of the untreated one. This increased fluorescence in case of *S. aureus* cells treated with peptide-Ba49 at 1 × MIC and 2 × MIC was due to the depolarization of the cytoplasmic membrane of the *S. aureus* (Figure 4).

**FIGURE 5:**
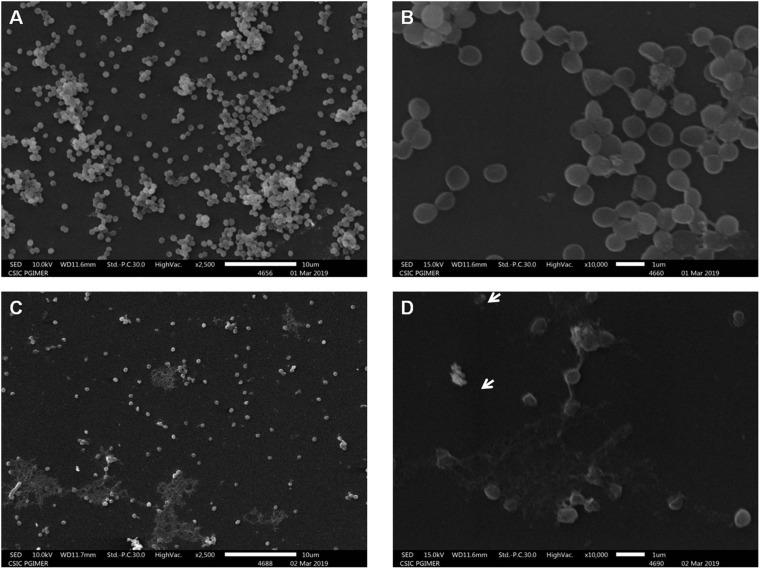
Scanning electron microscopy of *S. aureus* cells treated with purified peptide-Ba49. **(A,B)** Untreated *S. aureus* cells from the control group at 2.5 and 10 K resolution. **(C,D)** Purified peptide-Ba49-treated *S. aureus* cells at 2.5 and 10 K resolution. A white arrow shows ruptured cells after peptide treatment for a duration of 240 min.

Subsequent studies on cell morphology by SEM showed that the *S. aureus* cells without peptide-Ba49 treatment had a smooth surface (Figures 5A,B) as compared to some apparent morphological alterations (like damaging the cell envelope and destruction of the cells) in the case of cells treated with peptide-Ba49 (Figures 5C,D).

#### Evaluation of Inhibitory Effect of Peptide-Ba49 on *S. aureus* Biofilm Formation

**FIGURE 6:**
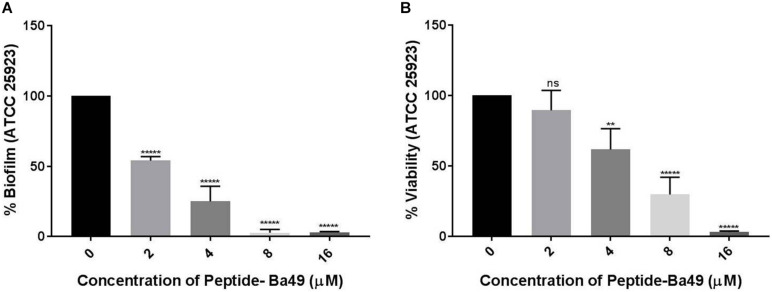
Effects of peptide-Ba49 on inhibition of *S. aureus* biofilm formation. **(A)** *S. aureus* biofilm biomass was measured using crystal violet assay. **(B)** Viability was measured by using XTT assay. One-way ANOVA followed by Dunnett’s test for multiple comparisons, *N* = 2 independent experiments with triplicates, **p < 0.01, *****p < 0.00001, and *p* > 0.05 were considered as non-significant (ns).

The effect of peptide-Ba49 on inhibition of *S. aureus* ATCC 25923 biofilm formation was evaluated at different concentrations of the peptide, i.e., 2, 4, 8, and 16 μM, as shown in Figure 6A. It could be observed that the biomass of *S. aureus* biofilm was significantly reduced in the presence of the peptide-Ba49. The peptide-Ba49 at concentrations of 8 and 16 μM inhibited about 90% of biofilm formation. Furthermore, the XTT assay was used to determine the viability of the cells in the biofilm, and as shown in Figure 6B, a significant reduction in viable cells within the biofilm in the presence of peptide-Ba49 was observed. At a peptide concentration of 8 μM, more than 50% of cells within the biofilm were viable, whereas at a concentration of 16 μM of peptide-Ba49, the cell viability was almost completely reduced.

#### CLSM of *S. aureus* Biofilm

**FIGURE 7:**
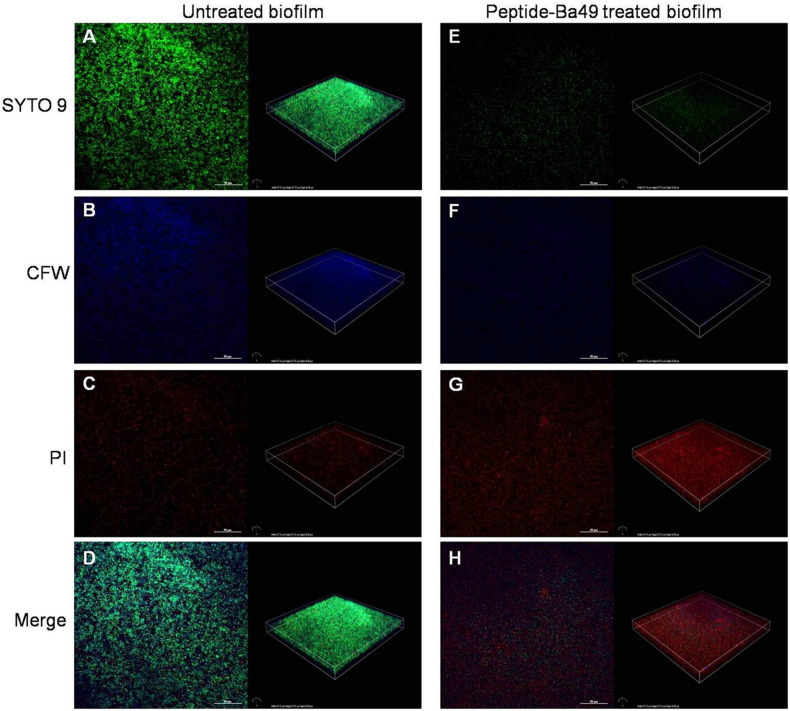
Confocal imaging of live/dead stained *S. aureus* biofilm [left—green channel (live), middle—red channel (dead), and right—merged channel]. 2D and 3D images showing the *S. aureus* biofilm inhibition effect of peptide-Ba49; untreated *S. aureus* biofilm **(A–D)** and Peptide-Ba49-treated biofilm **(E–H)** were post stained with Syto 9 (live stain, green), CFW (EPS stain, blue), and PI (dead stain, red).

CLSM studies showed that the peptide-Ba49-treated *S. aureus* biofilm could maintain the overall structure as compared to the untreated one. However, in the treated biofilm, the viable cells were less compared to the untreated biofilm (Figure 7). This could be due to the interaction of AMPs with the cytoplasmic membrane of the bacterial cells, causing the rupture of the cell membrane and leading to cell lysis (Bessa et al., 2019). Thus, in the peptide-treated biofilm, the red (PI labeled) cells are more as compared to the untreated one (Figures 7C,G). Calcofluor white M2R (CFW) dye was used to stain the EPS (extracellular polymeric substance), and a reduction in the blue signal was observed in the peptide-treated biofilm as compared to the untreated one (Figures 7B,F). This indicated that peptide-Ba49 prevented the biofilm formation of *S. aureus* by rupturing the cell membrane, and moreover, because of the low EPS formation, the adhesion to the surface was prevented.

#### Effect of the Peptide-Ba49 on Eradication of Biofilm

**FIGURE 8:**
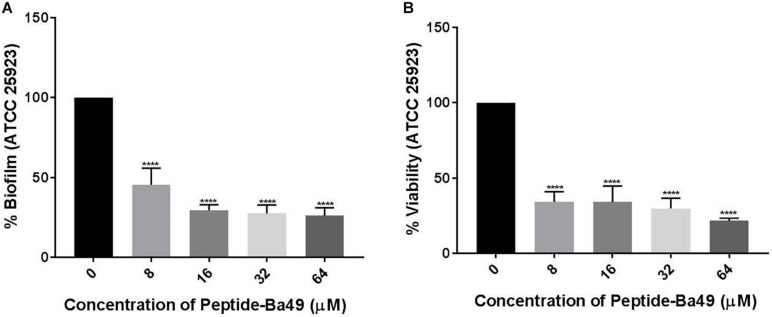
Effects of peptide-Ba49 treatment on inhibition of pre-formed biofilm of *S. aureus*. **(A)** Pre-formed *S. aureus* biofilm biomass after peptide-Ba49 treatment was estimated using crystal violet assay. **(B)** Viability was evaluated using XTT assay. One-way ANOVA followed by Dunnett’s test for multiple comparisons, *N* = 2 independent experiments with triplicates, ****p < 0.0001.

The effects of peptide-Ba49 on a mature *S. aureus* biofilm was studied by treating it with varied concentrations of the peptide and observing the reductive effect, followed by additional 24 h incubation. In the case of the mature biofilm, the number of bacteria was increased up to 10-fold; thus, higher concentrations of peptide-Ba49 were used to treat the mature biofilm, i.e., 8, 16, 32, and 64 μM. Treatment with peptide-Ba49 at concentrations of 16–64 μM resulted in a significant reduction in preformed biofilm biomass (OD_595_), as shown in Figure 8A. The colorimetric reduction assay of tetrazolium salt (XTT) also showed that about 80% of the cells in the biofilm were metabolically inactive at a concentration of 64 μM of peptide-Ba49 (Figure 8B).

#### Cell Migration Assay

**FIGURE 9:**
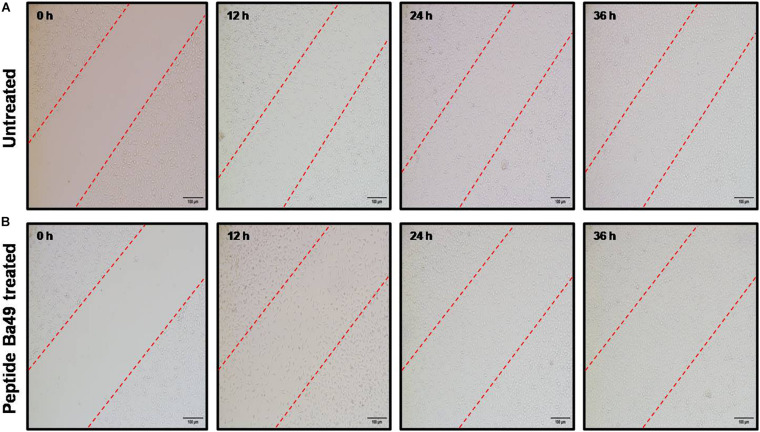
Cell migration assay. The fibroblast cells L929 were treated with peptide-Ba49 and observed under microscope until 36 h to evaluate the cell migration: **(A)** Untreated L929 cells. **(B)** Peptide-Ba49-treated L929 cells.

Cell migration is a rate-limiting factor in the wound healing process (Cappiello et al., 2018). The migration capacity of the murine fibroblast cells (L929) under the stimulation of peptide-Ba49 was determined by scratch assay to evaluate wound closure *in vitro*. It was observed that after the stimulation of L929 cells with peptide, the migration capacity became faster and the wound closure was approximately 50% at the 24 h time point. Moreover, following 36 h of treatment of the L929 fibroblast cell line with peptide-Ba49, almost complete wound closure was observed as compared to control, i.e., untreated cells (Figure 9).

## Discussion

The ESKAPE pathogens (*Enterococcus faecium*, *S. aureus*, *Klebsiella pneumoniae*, *Acinetobacter baumannii*, *Pseudomonas aeruginosa*, and *Enterobacter* species) are the most resistant to almost all the antibiotics and cause various chronic infections. *S. aureus* is one of the pathogens that infect the host and cause mild to chronic infections (Haddadin et al., 2002; Koch et al., 2014). Furthermore, the alarming factor related to *S. aureus* infection is their capability to form biofilm and become highly resistant to host attacks and antimicrobials (Lewis, 2001; Mah and O’toole, 2001; Savage et al., 2013; Koch et al., 2014). The biofilm cells are generally more than 1,000 times resistant to planktonic cells though it may vary from organism to organism. Various factors affect biofilm resistance, such as growth rate, temperature, nutritional value, and pH, and the most critical factor affecting biofilm resistance is the slower diffusion of drugs (Shikha et al., 2020). This necessitates the urgency for antibiotics that not only eradicates the planktonic cells but also has the potential to decimate the biofilm. *S. aureus* is also termed as a facultative intracellular pathogen and has the ability to survive within the host cells and escape the detection of professional phagocytes (Fraunholz and Sinha, 2012). Therefore, the intracellular bacteria enhance drug resistance development by protecting it from a higher concentration of extracellular antibiotics (Thwaites and Gant, 2011), thereby necessitating a need to develop new strategies to combat infectious pathogens. In that direction, AMPs are a new treatment strategy for bacterial infections that are rapidly in focus due to increased resistance to conventional antibiotics (Pfalzgraff et al., 2018).

In our previous study, an AMP, i.e., peptide-Ba49, was isolated and purified from *B. subtilis* subsp. *spizizenii* Ba49 (MTCC 13006), a strain isolated from *Allium cepa*. Later, based on whole genome analysis and *de novo* amino acid sequencing, the peptide-Ba49 was found to be identical to subtilin and shown to be having low MIC values in the range of 0.5–16 μM against different *Staphylococcus* strains and MRSA strains. Subsequently, the time kill studies showed a fast bactericidal efficacy against S. *aureus*, i.e., within 4 h (Taggar et al., 2021). In the present study, the role of purified peptide-Ba49 against methicillin-sensitive *S. aureus* ATCC 25923 strain was further deciphered. The potential of this peptide as a therapeutic candidate was evaluated by studying its PAE and *in vitro* intracellular infection. PAE is defined as the suppression period of bacterial growth that persists after a limited exposure of organisms to antimicrobials (Nedbalcova et al., 2019), and the degree of PAE relates to the degree of cellular damage done by the antimicrobial agent to the bacterial cell (Haukland and Vorland, 2001). The PAE of peptide-Ba49 indicated an extended PAE, i.e., 10 h against *S. aureus* after 4 h of exposure at a concentration of 8 μM (2 × MIC) (Figure 1B). This suggested that the damage to the cell could be immense and might take a long time to repair. In comparison, a naturally produced AMP DLP4 from hemolymph of *Hermetia illucens* has been reported to have a PAE value of 8.83 h against *S. aureus* (Li et al., 2020), and also a synthetic AMP, LTX-109, was shown to have 5.51 h of PAE against *S. aureus* (Saravolatz et al., 2017).

It is also well known that *S. aureus* can penetrate and survive within the host cell and cause chronic infections. Due to the difficulty in antibiotic passaging through cellular membranes, it becomes more challenging to treat the infection at the intracellular stage. So, in addition to the extracellular activity of the AMP, it is also required to have intracellular killing activity (Wang et al., 2018). Previously, it was shown that the peptide-Ba49 has low cytotoxic effect on the various cell lines (Taggar et al., 2021). The investigations on the intracellular killing activity of peptide-Ba49 showed a significant reduction in bacterial burden upon treating *S. aureus*-infected macrophages with peptide-Ba49.

Furthermore, the mechanistic insight into peptide-Ba49-mediated killing of *S. aureus* was elucidated. The intracellular generation of ROS was enhanced after the treatment with the peptide at a concentration of 8 μM, as measured by using a ROS-sensitive probe, H_2_DCFDA. It suggested that intracellular production of ROS increased the oxidative environment of the cell by altering the cell membrane resting potential. Moreover, oxidative stress plays a crucial role in altering the bacterial membrane permeability and damage the cell membranes (Shaikh et al., 2019). Furthermore, a voltage-sensitive DiSC_3_ (5) probe was used to investigate the change in *S. aureus* cell membrane permeability after treatment of peptide-Ba49. Interestingly, an increase in fluorescence was observed, indicating a change in the membrane potential of *S. aureus* cells after peptide treatment. This was further confirmed by morphological changes analyzed by SEM, which showed membrane lesions (Figure 5D), indicating that the damage of the cell could have occurred by change in membrane potential of *S. aureus* cells following peptide treatment.

*S. aureus* biofilm infections are of a primary concern compared to planktonic cell infections, and this is also a cause for resistance to conventional antibiotics (Savage et al., 2013; Koch et al., 2014; Olsen, 2015). An infection associated with biofilm requires high drug concentrations and are extremely difficult to treat (Koch et al., 2014; Mcconoughey et al., 2014; Liu et al., 2015). Interestingly, it was found that the peptide-Ba49 could inhibit *S. aureus* biofilm formation at a concentration of 8 and 16 μM within 24 h. The confocal imaging using PI and SYTO-9 showed the inhibition of biofilm formation treated with peptide-Ba49 (Figures 7E,G) compared to the control, i.e., untreated cells (Figure 7F). This was further confirmed by less EPS production during biofilm formation in the presence of peptide-Ba49 as compared to control (Figure 7B), indicating inhibition of *S. aureus* growth in the presence of peptide-Ba49. However, at a concentration of 16 μM, there was a significant reduction in viability of bacterial cells within the biofilm. Peptide-Ba49 was also found to kill the bacterial cells in the mature biofilm of *S. aureus* ATCC 25923 after 24 h of peptide treatment of *S. aureus* cells at a concentration of 64 μM where the cell biomass and cell viability within the mature biofilm were significantly reduced. These results signified that peptide-Ba49 could be developed as an antimicrobial agent for treating *S. aureus* planktonic as well as *S. aureus* biofilm-associated infections.

In the wound healing phase, cell proliferation and migration are two essential features, whereas an *in vitro* scratch assay mimics a wound and evaluates the cell migration rate. Due to the disruption of the cell monolayer, it loses cell–cell interaction, and increasing concentration of growth factors and cytokines at the edge of the wound further initiates cell migration and proliferation (Liang et al., 2007; Pitz Hda et al., 2016). Interestingly, peptide-Ba49 at a concentration of 2 × MIC prompted the L929 fibroblast cell proliferation. This is a positive event for the wound healing process, as fibroblast cells are essential cells because of their involvement in wound contraction and ECM production (Balekar et al., 2012).

**FIGURE 10:**
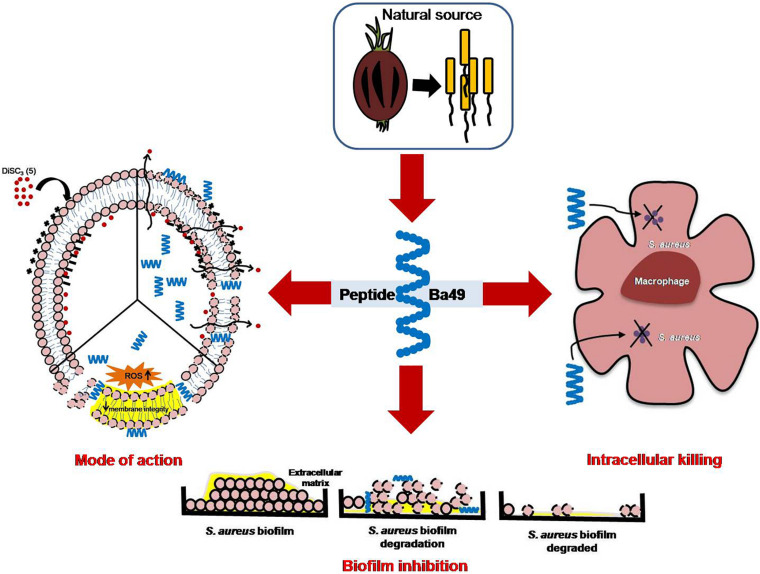
Proposed mechanism of action of peptide-Ba49 isolated from *Bacillus subtilis* subsp. *spizizenii* strain from *Allium* cepa against *S. aureus*.

In conclusion, the peptide-Ba49 showed antimicrobial activity against MRSA isolate. This peptide was effective against *S. aureus* planktonic cells and showed pre- and post-antibiofilm activity at low concentration. It also demonstrated intracellular activity in the MIC range studied and efficiently reduced the bacterial burden from *S. aureus*-infected macrophages (RAW264.7). All these properties of peptide-Ba49 established it as a potential candidate for therapeutic applications (Figure 10).

## Data Availability Statement

The original contributions presented in the study are included in the article/Supplementary Material, further inquiries can be directed to the corresponding author.

## Author Contributions

DS, RT, and SS conceived the project. RT and SS performed the experiments. DS, RT, and SS analyzed the data, wrote and edited the manuscript, with input from all of the authors. All authors contributed to the article and approved the submitted version.

## Conflict of Interest

The authors declare that the research was conducted in the absence of any commercial or financial relationships that could be construed as a potential conflict of interest.

## Publisher’s Note

All claims expressed in this article are solely those of the authors and do not necessarily represent those of their affiliated organizations, or those of the publisher, the editors and the reviewers. Any product that may be evaluated in this article, or claim that may be made by its manufacturer, is not guaranteed or endorsed by the publisher.
